# Popliteal Artery Injury After Arthroscopic Knee Surgery: A Retrospective Multicenter Cohort Study

**DOI:** 10.1111/os.14334

**Published:** 2024-12-30

**Authors:** Zhenmu Xu, Kai Jiang, Yueming Chen, Hao He, Weihong Zhu

**Affiliations:** ^1^ Department of Orthopaedics The Second Xiangya Hospital of Central South University Changsha Hunan China; ^2^ Department of Orthopaedics The First Hospital of Hunan University of Chinese Medicine Changsha Hunan China; ^3^ Department of Orthopaedics Central Hospital of Shaoyang Shaoyang Hunan China; ^4^ Department of Vascular Surgery, The Second Xiangya Hospital Central South University Changsha China; ^5^ The Institute of Vascular Diseases Central South University Changsha China

**Keywords:** arthroscopic knee surgery, endovascular treatment, popliteal artery injury

## Abstract

**Objective:**

Popliteal artery injury is a rare but serious complication of arthroscopic knee surgery. The absence of comprehensive data and standardized guidelines underscores the urgent need for further investigation. This study examines the incidence, risk factors, management strategies, and long‐term outcomes of popliteal artery injury in the context of arthroscopic knee procedures.

**Methods:**

We conducted a retrospective cohort study utilizing data from 21 medical institutions in Hunan Province, China, from January 2018 to December 2022. We identified patients who underwent arthroscopic knee surgery and complained of postsurgical popliteal artery injury. Patients were followed up for 43.1 ± 13.23 months (ranging from 22 to 58 months). The primary outcome was joint function, which was evaluated by a postoperative range of motion (ROM), International Knee Documentation Committee (IKDC) scores, Lysholm knee scores, and Visual Vascular Quality of Life Questionnaire (VascuQoL) scores. These data from different postoperative periods were compared via paired *t*‐test to assess postoperative recovery. The secondary outcome was vascular patency of the affected limb, which was evaluated through vascular color Doppler ultrasound.

**Results:**

Among the 17,000 knee arthroscopic procedures analyzed, 10 patients were identified with popliteal artery injury (0.059%). The surgeries performed included arthroscopic cystectomy for popliteal cysts, cruciate ligament reconstruction, and posterior horn of the lateral meniscus repair. Treatments for popliteal artery injury included percutaneous intravascular stent implantation (one patient), direct suture repair (four patients), allograft vascular transplantation (one patient), and reconstruction with an autogenous greater saphenous vein (four patients). After a mean follow‐up time of 43.1 ± 13.23 months (ranging from 22 to 58 months), no complications were reported. Compared with patients at 1 month after surgery, patients at 2 years after surgery presented improved knee function and ROM. The average Lysholm score increased significantly from 13.8 ± 4.21 to 68.2 ± 15.50, the IKDC score increased from 11.6 ± 2.46 to 48.1 ± 11.75, and the VascuQoL score improved from 54.8 ± 9.54 to 92.5 ± 15.90. Knee extension improved from 13.3° ± 2.36° to 3.5° ± 4.12°, and knee flexion increased from 49.5° ± 12.57° to 107° ± 21.63°. All patients successfully resumed daily activities postoperatively.

**Conclusion:**

Popliteal artery injury is a catastrophic complication that warrants significant attention during knee arthroscopy. This injury can occur in various types of arthroscopic knee procedures. Prompt diagnosis and effective intervention are crucial for minimizing the potential detriment associated with popliteal artery injury.

## Graphical abstract

In a retrospective study of 17,000 knee arthroscopic procedures, the incidence of popliteal artery injury was found to be 0.059%. Timely and appropriate vascular interventions were crucial for recovery, significantly enhancing knee function, range of motion, and quality of life over a follow‐up period of 43.1 ± 13.23 months. Early recognition and suitable vascular repair are essential to manage this rare yet severe complication.

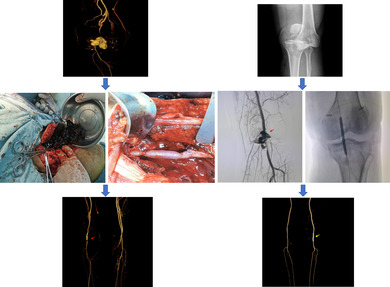

## 1 Introduction

Arthroscopic knee surgery is one of the most common orthopedic procedures. Although it is considered minimally invasive, it carries the risk of serious complications [1, 2]. Postarthroscopic complications have been reported to occur at rates ranging from 0.8% to 8.2%, with some patients experiencing restricted ranges of movement, infections, and neurovascular injuries [3]. Vascular complications, although exceptionally rare, can have catastrophic consequences if they occur during arthroscopic knee surgery, particularly when major vessels are involved [4, 5, 6]. Arterial injuries commonly manifest as intraoperative bleeding, acute ischemia with diminished peripheral pulses, persistent wounds, and limb swelling [7, 8, 9, 10]. These situations can potentially result in irreversible ischemia, permanent nerve damage, compartment syndrome, amputation, and even death.

Owing to its close proximity to the posterior joint capsule, posterior cruciate ligament, and lateral meniscus posterior horn, the popliteal artery is especially at risk of injury during arthroscopic knee surgery. In addition, popliteal artery injury can also occur in other procedures, such as knee arthroplasty or osteotomy [11, 12, 13, 14]. Despite its rarity, a delayed diagnosis can lead to devastating outcomes. Therefore, prompt exploration and repair are crucial in the context of vascular injury. However, methods for preventing and managing vascular injuries during both the preoperative and intraoperative phases have not reached a unified consensus.

The objectives of this study are: (i) to analyze the incidence and causes of popliteal artery injury during arthroscopic knee surgeries in Hunan Province over the past 5 years, (ii) to provide preventive measures to reduce the incidence of popliteal artery injury during arthroscopic knee surgery in the future, and (iii) to suggest strategies for managing popliteal artery injury.

## 2 Materials and Methods

### 2.1 Demographics and Data Collection

We conducted a multicenter retrospective cohort study in which data from arthroscopic knee procedures performed at 21 medical institutions in Hunan Province, China, were collected between January 2018 and December 2022. This study was approved by the Second Xiangya Hospital Committee for clinical research (LYF20240161). The inclusion criteria were (i) arthroscopic knee surgeries performed within the last 5 years and (ii) no previous arterial injury or vascular surgery. The exclusion criteria included (i) incomplete records of surgical procedures and follow‐up data and (ii) patients with a history of alternative knee joint surgeries.

Data on arthroscopic knee surgeries were collected from all participating institutions through questionnaire surveys, representing a sample from the province over the past 5 years. Cases involving popliteal artery injury were identified on the basis of these data. For these patients, comprehensive medical history records, physical examination notes, imaging data (including MRI, CTA, and x‐ray), types of arthroscopic procedures performed, and management strategies for popliteal artery injuries were systematically gathered. Patients were evaluated at 1, 3, 6, 12, 24, and 48 months postoperatively. During each follow‐up visit, assessments included knee range of motion (ROM), the Lysholm score, the International Knee Documentation Committee (IKDC) score, and the Visual Vascular Quality of Life Questionnaire (VascuQoL). In addition, lower limb vascular status was examined via color Doppler ultrasound.

### 2.2 Statistical Analyses

All the statistical analyses were performed via SPSS 24.0 software (IBM Corp., Armonk, NY, USA). The baseline characteristics of the patients are presented as numbers for categorical data and as the mean ± standard deviation for continuous data. A paired *t*‐test was used to compare continuous data; *p* < 0.001 was considered to indicate statistical significance.

## 3 Results

### 3.1 Incidence of Popliteal Artery Injury

We identified 10 cases of popliteal artery injury from over 17,000 surgeries, with an average patient age of 43.9 ± 9.27 years (range: 33–64 years). The incidence of popliteal artery injury in arthroscopic knee surgeries performed in Hunan Province over the past 5 years has been relatively low, at approximately 0.059%. Table 1 presents the demographic characteristics of the affected patients and the distribution of popliteal artery injury by type of arthroscopic procedure.

**TABLE 1:** Demographic data of all consecutive patients.

Demographic variable	Mean or proportion
Age (years)	43.90 ± 9.27 (33–64)
BMI (kg/m^2^)	22.92 ± 4.31 (16.55–31.87)
Sides, *n* (%)	
Left	4 (40%)
Right	6 (60%)
Gender, *n* (%)	
Female	5 (50%)
Male	5 (50%)
Discovery time, *n* (%)	
Intraoperative	8 (80%)
Postoperative	2 (20%)
Procedure, *n* (%)	
Arthroscopic ACLR and PCLR	4
Arthroscopic PCLR	4
Arthroscopic popliteal cyst excision	1
Arthroscopic lateral meniscus suture	1
Treatment after artery injury, *n* (%)	
Percutaneous intravascular stent implantation	1
Direct suture	4
Allograft vascular transplantation	1
Reconstruction with autogenous greater saphenous vein	4

*Note*: Data are presented as percentage (%) or mean ± SD.

Abbreviations: ACLR, anterior cruciate ligament reconstruction; *n*, number of individuals; PCLR, posterior cruciate ligament reconstruction.

### 3.2 Types of Arthroscopic Procedures and Popliteal Artery Injuries

The surgeries performed included arthroscopic cystectomy for popliteal cysts, anterior cruciate ligament reconstruction (ACLR), posterior cruciate ligament reconstruction (PCLR), and posterior horn of the lateral meniscus repair. The distribution of popliteal artery injury by surgical procedure was as follows: three cases occurred during arthroscopic ACLR and PCLR, three during arthroscopic PCLR, one during arthroscopic popliteal cyst excision, and one during arthroscopic lateral meniscus repair.

### 3.3 Clinical Presentation and Diagnosis

Among these patients, one had a nonpalpable dorsalis pedis artery after arthroscopic ACLR and PCLR. CTA revealed a popliteal artery embolism, prompting immediate vascular exploration and repair. Another patient, identified 1 month after arthroscopic PCLR, presented with a gradually enlarging mass in the popliteal fossa. CTA indicated a pseudoaneurysm, and the patient underwent popliteal artery pseudoaneurysm resection and reconstruction using an autogenous greater saphenous vein. In the remaining patients, significant intra‐articular bleeding that could not be controlled was observed during surgery, leading to suspension of the procedure. Immediate CTA confirmed popliteal artery injury in these patients.

### 3.4 Management and Surgical Interventions

All patients received timely vascular surgical interventions, including percutaneous intravascular stent implantation (one patient), direct suture repair (three patients), allograft vascular transplantation (one patient), and reconstruction with an autogenous greater saphenous vein (three patients). In one patient with multiple ligament injuries, the popliteal artery injury was discovered after the completion of ACL and PCL reconstruction. Vascular exploration and repair were immediately performed, and the lateral collateral ligament repair was terminated. In the other cases, when the popliteal artery injury was identified during the operation, the surgical team promptly paused the primary procedure to prioritize artery repair or reconstruction, ensuring that the patient's condition stabilized before proceeding with the remaining surgical steps.

### 3.5 Postoperative Management and Follow‐Up

Following surgery, all 10 patients underwent postoperative CTA examinations, confirming unobstructed blood flow at the site of the popliteal artery injury, with no evidence of blood leakage, thrombosis, or other complications. Patients received anticoagulation therapy for 3 months postoperatively, followed by a transition to antiplatelet therapy on the basis of individual circumstances. Knee immobilization is necessary after surgery to prevent excessive flexion and rehabilitation exercises such as ankle pumps are recommended to prevent deep vein thrombosis. In addition, all patients underwent follow‐up lower limb vascular color Doppler ultrasound examinations.

The mean follow‐up time was 43.1 ± 13.23 months (range: 22–58 months), and the final follow‐up confirmed that all patients had unobstructed blood flow in the popliteal artery, without thrombosis, infection, or aneurysm formation at the anastomosis site.

### 3.6 Knee Function and ROM at 2‐Year Follow‐Up

At the 2‐year follow‐up, patients showed significant improvements in knee function scores and ROM. Compared with those at the 1‐month postoperative evaluation, the Lysholm score increased from 13.8 ± 4.21 to 68.2 ± 15.50, the IKDC score rose from 11.6 ± 2.46 to 48.1 ± 11.75, and the VascuQoL score improved from 54.8 ± 9.54 to 92.5 ± 15.90.

In terms of ROM, the average knee extension improved from 13.3° ± 2.36° at 1 month to 3.5° ± 4.12° at 2 years, and the average knee flexion increased significantly from 49.5° ± 12.57° at 1 month to 107° ± 21.63° at 2 years. The detailed clinical data are presented in Table 2.

**TABLE 2:** Comparison of knee function scores at 1 month and 2 years postoperatively.

Evaluation item	Postoperative 1 month	Postoperative 2 years	*p*
ROM			
Extension (°)	13.3 ± 2.36	3.5 ± 4.12	< 0.001
Flexion (°)	49.5 ± 12.57	107 ± 21.63	< 0.001
Lysholm score	13.8 ± 4.21	68.2 ± 15.50	< 0.001
IKDC score	11.6 ± 2.46	48.1 ± 11.75	< 0.001
VascuQoL score	54.8 ± 9.54	92.5 ± 15.90	< 0.001

*Note*: Data are mean ± SD.

### 3.7 Postoperative Complications and Long‐Term Outcomes

At the last follow‐up, patients who underwent allograft vascular reconstruction reported numbness in the dorsum of the foot. Two patients who underwent direct vessel suturing experienced swelling and mild discomfort in the popliteal fossa. The patient who underwent popliteal artery pseudoaneurysm resection and reconstruction with an autogenous greater saphenous vein exhibited mild swelling and limited knee flexion on the affected side. In addition, one patient who underwent reconstruction with the greater saphenous vein lost a significant portion of motor and sensory functions below the knee joint. However, the remaining patients experienced no significant discomfort or notable restrictions in daily activities. Notably, none of these patients underwent amputation or died postoperatively due to limb ischemia.

## 4 Case Presentation

We present two instructive cases and commonly used surgical methods.

### 4.1 Case 1

**FIGURE 1:**
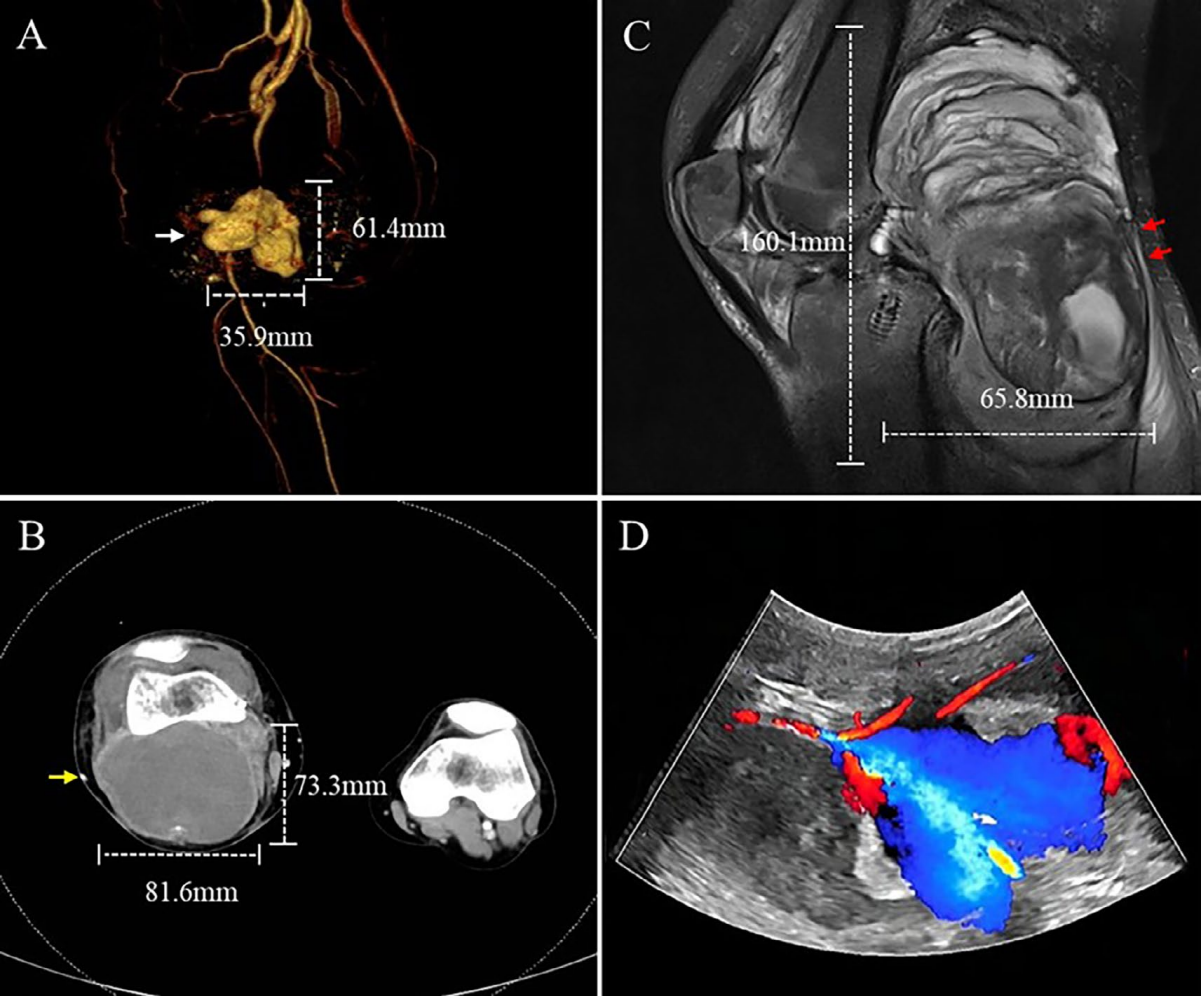
Preoperative imaging results of the patient. (A) Preoperative CTA revealed a popliteal mass measuring approximately 61.4 × 35.9 mm (white arrow). (B) An axial view of the preoperative CTA showed an occupancy of approximately 73.3 × 81.6 mm (yellow arrow). (C) Preoperative MRI showed a clear‐cut mass‐like mixed signal in the popliteal fossa with a cross‐sectional area of approximately 160.1 × 65.8 mm (red arrow). The signal intensity of the PCLR graft is mixed. (D) Color Doppler ultrasound of the lower extremity vessels showed a mixed echogenic mass in the popliteal fossa.

A 38‐year‐old female underwent PCLR of the right knee 2 months ago because of a traffic accident. One‐month post‐surgery, the patient developed a gradually enlarging popliteal mass, accompanied by swelling and elevated skin temperature. Ultrasound revealed a lesion of approximately 6 cm on the ventral side of the popliteal artery, in line with the tibial canal, and a lobed pseudoaneurysm. Physical examination revealed a painful, tense, and swollen calf with weak dorsalis pedis artery pulsation. The ankle‐brachial ratio (ABI) of the right lower extremity was 0. The ROM was limited to 30° of extension and 60° of flexion. The results of the reverse Lachman test and posterior drawer test were negative. The laboratory test was negative. CTA revealed a large pseudoaneurysm of the popliteal artery (Figure 1A,B). MRI confirmed that there were hyperintense and mixed signals behind the PCLR graft and a hypointense signal occupying the lesion of the popliteal fossa (Figure 1C). Color Doppler ultrasound of the lower extremity vessels revealed a mixed echogenic mass in the popliteal fossa (Figure 1D).

**FIGURE 2:**
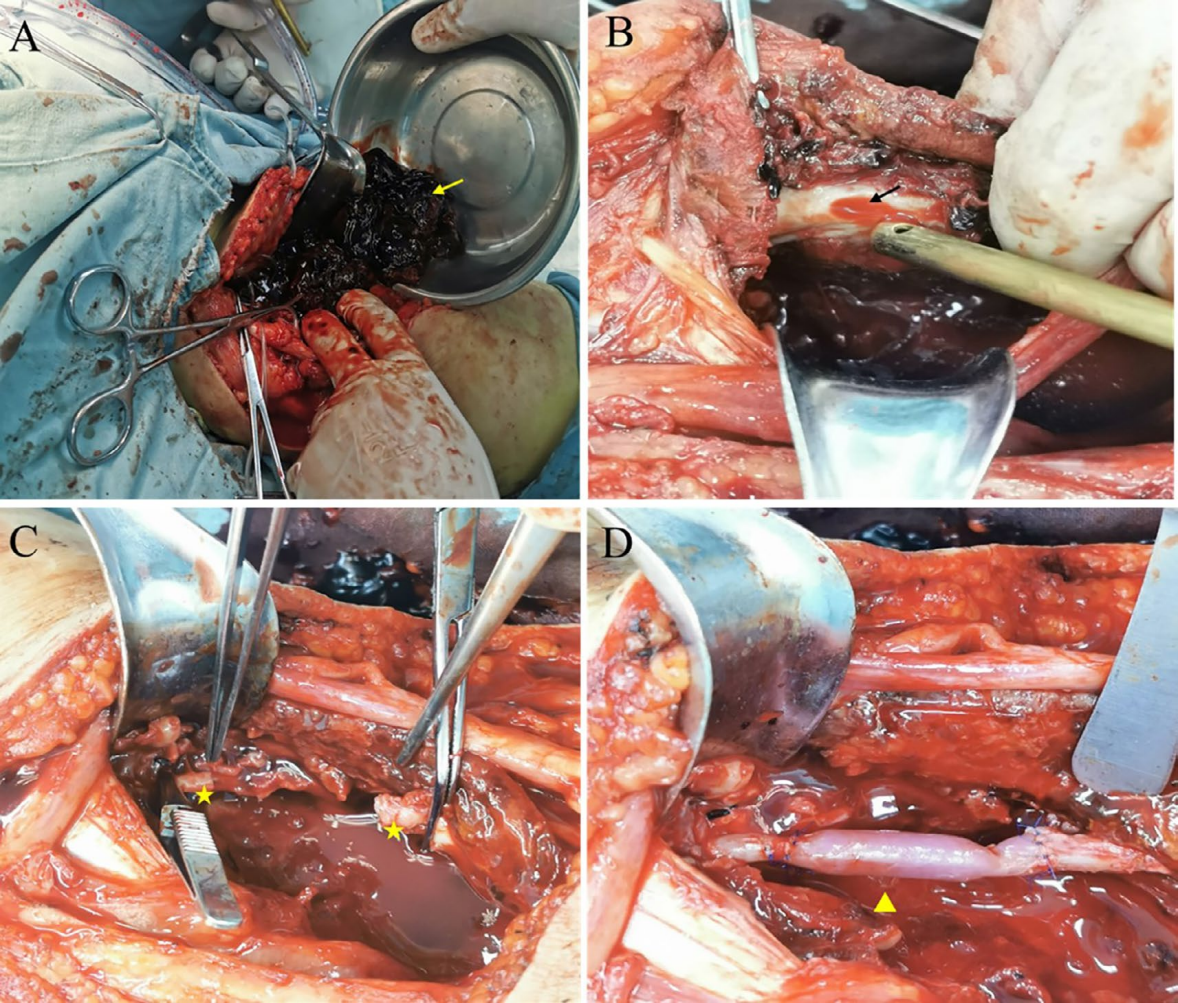
Intraoperative photos of this patient. (A) Many black blood clots (yellow arrow) were visible at the incision of the popliteal fossa. (B) An obvious rupture in the middle section of the popliteal artery (black arrow) was visible after the blood clots were removed. (C) After removing the torn popliteal artery, the distal and proximal ends of the popliteal artery (yellow pentagram) were dissected freely. (D) The great saphenous vein (yellow triangle) was grafted over to anastomose the two severed ends.

An S‐shaped incision was dissected, starting from the medial side of the biceps femoris to 10 cm above the vastus medialis. Upon retracting the medial head of the gastrocnemius and the semimembranosus muscles medially and the lateral head of the gastrocnemius laterally, a large, high‐tension mass was found in the posterior compartment of the popliteal fossa with no pulsation. Exploration revealed a lesion approximately 2 cm in length with rough edges on the ventral side of the popliteal artery. Owing to the nature of the lesion, an end‐to‐end anastomosis of the popliteal artery was unable to proceed. A segment of the saphenous vein from the ipsilateral limb was harvested, and an end‐to‐end anastomosis was performed from the distal to the proximal end of the popliteal artery via a 6‐0 Prolene suture (Figure 2). Intraoperative assessment revealed that the anastomosis was patent, with no obvious blood leakage or stenosis, and that the artery was pulsating well both distally and proximally. Finally, the incision was closed in layers.

**FIGURE 3:**
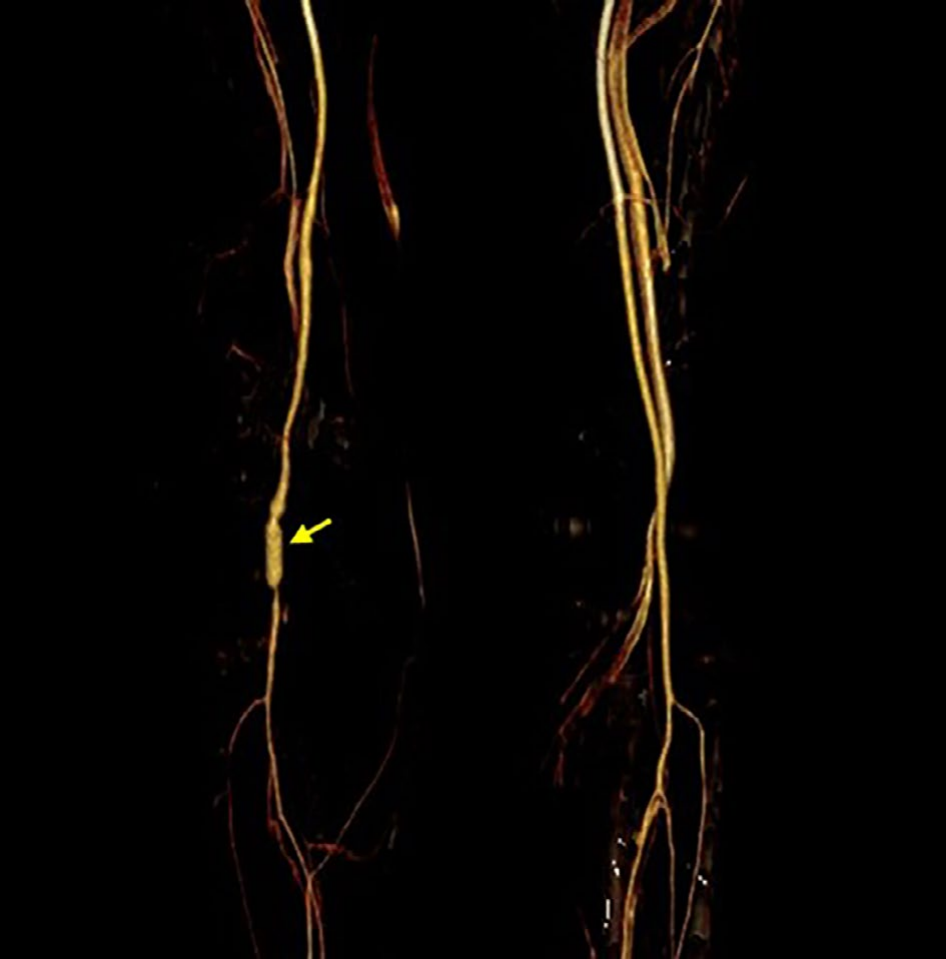
Postoperative CTA showed the patency of blood flow at the site of the great saphenous vein transplantation (yellow arrow).

Anticoagulant therapy was administered. Postoperative CTA indicated that the popliteal artery pseudoaneurysm had disappeared and that the popliteal artery was patent (Figure 3). At the 2‐year follow‐up, the patient exhibited only mild swelling and limited knee flexion on the affected side. The Lysholm score, which was 14 at the 1‐month postoperative evaluation, improved to 64 at the 2‐year postoperative evaluation. Similarly, the IKDC score improved from 11 to 41, and the VascuQoL score improved from 49 to 95 over the same period.

### 4.2 Case 2

A 64‐year‐old male with a history of a motorcycle accident was referred to a regional trauma center after being diagnosed with left knee dislocation, which was managed with manipulative reduction and plaster support. Physical examination revealed positive results for the anterior drawer, posterior drawer, floating patella, medial stress, and Lachman tests. The patient experienced longitudinal percussion pain in the lower extremity, numbness, and knee dysfunction, but the pulse of the dorsalis pedis artery was palpable.

**FIGURE 4:**
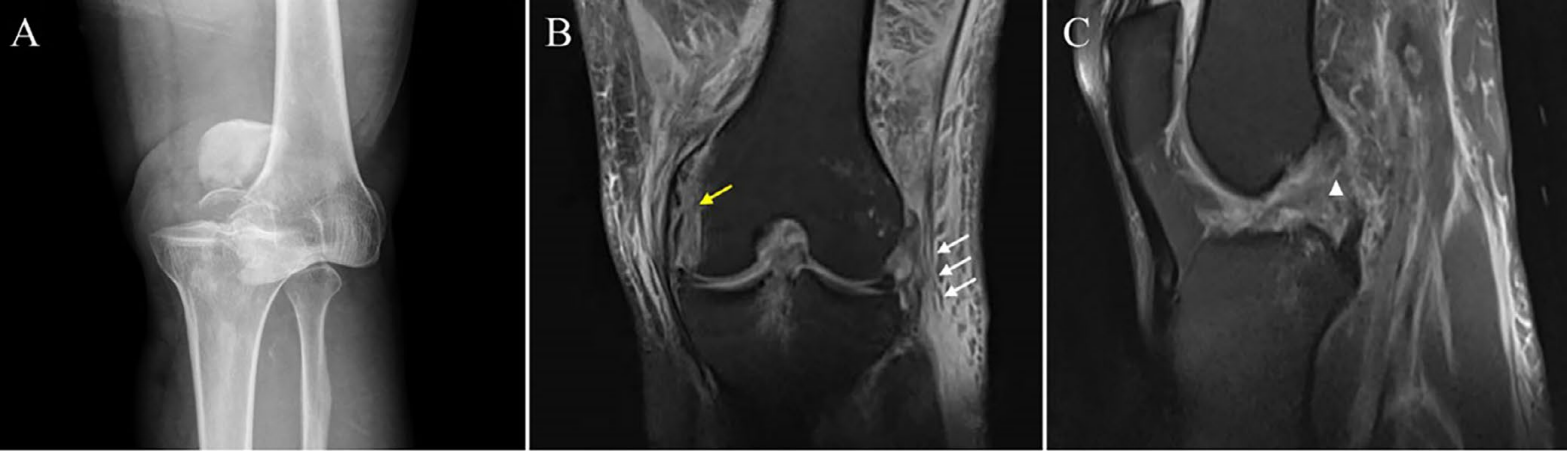
Preoperative imaging results of the patient. (A) Plain radiographs showed the affected knee joint before the reduction of left knee dislocation. (B) MRI indicated injuries to both the lateral collateral ligament (white arrow) and the medial collateral ligament (yellow arrow). (C) MRI showed the disruption of the continuity of the ACL and PCL (white triangle).

A plain radiograph revealed significant posterior displacement of the knee joint (Figure 4A). MRI further confirmed multiple ligament injuries, including those to the ACL, PCL, medial collateral ligament, and posterolateral complex (Figure 4B,C).

**FIGURE 5:**
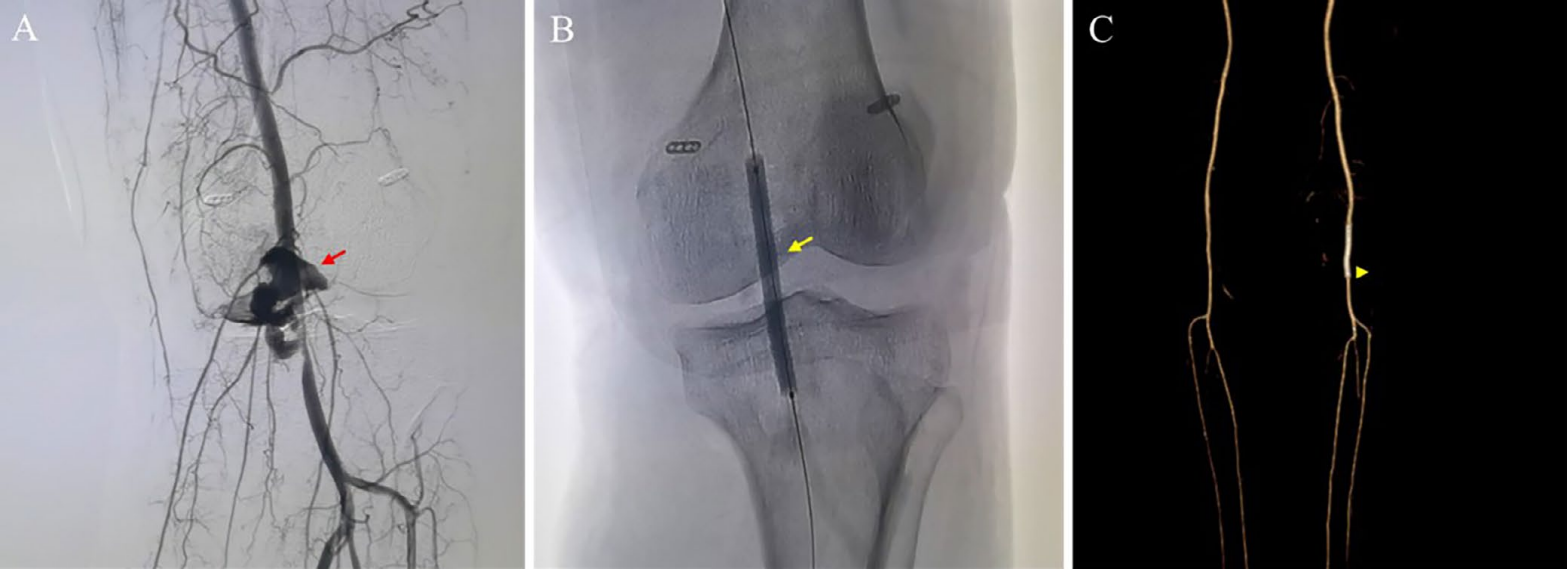
Imaging results of the patient. (A) DSA showed a ruptured popliteal artery pseudoaneurysm (red arrow). (B) A Viabahn Stent Graft was deployed and ballooned at the broken ends of the popliteal artery. (C) Postoperative CTA showed the patency of blood flow in the left popliteal artery (yellow arrow).

The patient underwent multiple ligament reconstructions 2 weeks after the injury. Arthroscopic inspection revealed that the ACL and PCL were completely ruptured. ACL and PCL reconstructions were performed successively. The posteromedial portal was established before the tibial socket of the PCL was drilled to avoid damaging the popliteal neurovascular bundle. After PCL graft fixation, warm blood poured from the ACL tibial tunnel, indicating possible popliteal artery damage. Attempts to stop the bleeding with radiofrequency failed, prompting consultation with a vascular surgeon. Digital subtraction angiography (DSA) revealed a popliteal artery pseudoaneurysm with a neck of 1 cm in diameter (Figure 5A). A Viabahn Stent Graft (5 mm × 6 cm; Gore, USA) was deployed (Figure 5B) and ballooned with a 6 mm × 8 cm Abbott balloon (Abbott Fox Plus PTA Dilatation, Abbott Park, IL, USA) in the popliteal artery. Postoperative imaging (Figure 5C) confirmed the exclusion of the aneurysm and preservation of the distal tibial vessels.

Postoperative care included blood transfusion and routine anticoagulation therapy. At the 2‐year follow‐up, the knee achieved full ROM and good stability. The Lysholm score, which was 18 at the 1‐month postoperative evaluation, improved to 74 at the 2‐year postoperative evaluation. Similarly, the IKDC score improved from 12 to 54, and the VascuQoL score improved from 68 to 103 over the same period.

## 5 Discussion

Our findings revealed that although popliteal artery injuries occur infrequently (0.059% in this cohort), their consequences, if untreated, can be severe, including limb ischemia and permanent functional impairment. Importantly, all patients in this study who received prompt vascular surgical intervention such as direct suture repair, stent implantation, or vascular reconstruction achieved favorable long‐term outcomes, with no reported complications during the follow‐up period. These results highlight the critical role of early detection and timely intervention in preserving limb function and ensuring optimal recovery.

### 5.1 Risk Factors and Causes

Popliteal artery injury during arthroscopic knee surgery can result from various risk factors. Anatomically, the popliteal artery is typically located behind the lateral meniscus, lateral to the PCL, and in front of the popliteal vein and tibial nerve. It generally originates where the femoral artery passes through the adductor hiatus and splits into two branches: the anterior tibial artery and the posterior tibial artery [15]. However, various classifications have shown that the morphology of the popliteal artery can vary. The most commonly used classification, developed by Kim, Orron, and Skillman [16] in 1989, identifies three subtypes. In Type 2, the popliteal artery divides at or above the knee joint, occurring in approximately 1.2%–7.8% of cases [16, 17, 18]. This variation increases the risk of popliteal artery injury during posterior knee surgeries when the path of the artery is altered. In addition, the position of the popliteal artery is close to the posterior capsule, and the internal and external rotation of the knee joint will cause the blood vessels to move accordingly [19]. Matava, Sethi, and Totty [20] found that the mean distance between the PCL and popliteal artery on the sagittal plane of MRI was 5.4 ± 2.1 mm at a knee flexion angle of 0° and increased to 7.8 ± 3.6 mm at a knee flexion angle of 90°. The variability in the anatomical position of the popliteal artery and the specificity of the area in which it is located makes it more susceptible to iatrogenic vascular trauma during arthroscopic knee surgery.

### 5.2 Prevention Strategies and Surgical Approach

Despite the inherent risks, arthroscopic knee surgery is minimally invasive, and the occurrence of popliteal artery injury remains rare. Nevertheless, surgeons must be vigilant and take preventive measures to avoid such complications.

Several studies have reported various methods to prevent popliteal artery injury during arthroscopic knee surgeries. First, maintaining a clear surgical field is crucial. If necessary, posterolateral and posteromedial portals should be established to fully expose the tibial footprint of the PCL or the boundaries of the popliteal cyst [21, 22, 23]. Second, when tibial tunnel drilling is performed, the knee should be flexed to 90° or even 100° to increase the distance between the tibia and the neurovascular bundle, thereby reducing the risk of iatrogenic vascular trauma [20, 24, 25]. It is also advisable to use C‐arm fluoroscopy intraoperatively to assess the appropriate position of the tip of the Kirschner wire when establishing the tibial tunnel. When the Kirschner wire is close enough to penetrate the bone cortex of the proximal tibia, switching the head and tail of the wire can help avoid vascular injury. In addition, if arthroscopists choose to drill the tibial tunnel blindly, there is a risk that sharp surgical instruments may damage the popliteal artery.

In addition, many arthroscopists have modified surgical techniques to reduce the risk of iatrogenic vascular lesions. Franciozi et al. [26] reported that creating a lateral tibial tunnel for PCL reconstruction minimizes the probability of popliteal artery injury. Alentorn‐Geli et al. [27, 28] described drilling the tibial tunnel with an anteriorly directed inside‐out technique using a flexible pin and reamer through the posterolateral portal, which can reduce the chance of intraoperative popliteal artery injury. With respect to popliteal cysts, Kp et al. [29] suggested that during arthroscopic cystectomy, the shaver might inadvertently injure the posteromedial wall of the popliteal artery, resulting in the formation of a pseudoaneurysm. Therefore, caution is advised when performing arthroscopic cystectomy, especially when MRI indicates the close proximity of the popliteal artery to the cyst. Mao et al. [30] suggested that compared with the use of an anterolateral portal, the use of the all‐inside technique to repair the posterior horn of the lateral meniscus lowers the risk of popliteal artery injury when the repair is performed through an anteromedial working portal.

Performing CTA before surgery can aid in assessing the presence of popliteal artery trauma, particularly in patients with knee dislocation [31]. For example, in the second case, a pseudoaneurysm may have developed due to injury to the popliteal artery immediately after the motor accident. Unfortunately, preoperative CTA was not conducted. During the operation, limb movement and instrument manipulation possibly exacerbated the situation, leading to the rupture of the pseudoaneurysm.

In our case series, one patient underwent popliteal cystectomy, and one patient underwent lateral meniscus posterior horn repair. Popliteal artery injury during popliteal cystectomy may be attributed to factors such as concurrent osteoarthritis, inadequate exposure of the posterior compartment, difficulty in instrument entry, and unfamiliarity with the anatomy near the medial head of the semimembranosus tendon and gastrocnemius muscle. This emphasizes the importance of comprehensive preoperative evaluation and preparation for surgeons. The possible reason for popliteal artery injury during lateral meniscus posterior horn repair may be inadequate depth control of the all‐inside meniscus suturing devices and an incorrect suturing angle.

### 5.3 Management of Popliteal Artery Injury

When popliteal artery injury occurs during arthroscopic knee surgery, it is essential to implement appropriate treatment strategies for repairing the injured artery. Open surgical procedures, including direct suturing repair, covering of the arterial defect with a venous patch, interposition grafting, or bypass grafting, have long been considered the gold standard for treating vascular injuries [32, 33, 34]. In the literature concerning popliteal artery injury during knee joint surgeries, most surgeons tend to favor vascular reconstruction through reversing greater saphenous vein grafting, performing direct suturing repair, or covering arterial defects with vein patches. Some researchers also prefer synthetic grafts for vascular reconstruction [4]. In addition, patients in this situation should immediately receive anticoagulant therapy to reduce the risk of subsequent complications [35].

## 6 Strengths and Limitations

This study is one of the few investigations into popliteal artery injury during arthroscopic knee surgery across multiple centers, providing a broader perspective on the incidence and management of this rare complication. The comprehensive follow‐up data, which include knee function scores, ROM measurements, and vascular patency assessments, offer valuable insights into the short‐term recovery and outcomes for affected patients. However, there are also limitations to consider. First, there is variability in data collection and potential inconsistencies in follow‐up evaluations since not all patients were assessed by the same physician. In addition, the reported incidence of popliteal artery injury may be underestimated due to potential underreporting by physicians or patients being hesitant to disclose adverse outcomes. The small sample size and relatively short follow‐up period further limit the generalizability of our findings and the ability to assess long‐term outcomes and complications. Future studies should focus on larger sample sizes, prospective designs, and longer follow‐up durations to validate our findings and evaluate the long‐term efficacy and safety of management strategies for popliteal artery injury.

## 7 Conclusion

Popliteal artery injury is a rare but serious complication of knee arthroscopy. Early identification and appropriate management, including prompt vascular repair and postoperative care, are crucial to preventing limb loss and improving patient outcomes.

## Author Contributions

Zhenmu Xu and Kai Jiang wrote the original draft. Weihong Zhu and Hao He supervised the work and edited the manuscript. Yueming Chen reviewed and edited the manuscript. All authors have read and approved the article.

## Ethics Statement

This study was approved by the Second Xiangya Hospital Committee for clinical research (LYF20240161).

## Conflicts of Interest

The authors declare no conflicts of interest.

## Supporting information


**Data S1.** Clinical history table.

## References

[1] T. Hagino , S. Ochiai , Y. Watanabe , et al., “Complications After Arthroscopic Knee Surgery,” Archives of Orthopaedic and Trauma Surgery 134 (2014): 1561–1564. 25047161 10.1007/s00402-014-2054-0

[2] S. G. F. Abram , A. Judge , D. J. Beard , H. A. Wilson , and A. J. Price , “Temporal Trends and Regional Variation in the Rate of Arthroscopic Knee Surgery in England: Analysis of Over 1.7 Million Procedures Between 1997 and 2017. Has Practice Changed in Response to New Evidence?,” British Journal of Sports Medicine 53 (2019): 1533–1538. 30279217 10.1136/bjsports-2018-099414

[3] M. J. Salzler , A. Lin , C. D. Miller , S. Herold , J. J. Irrgang , and C. D. Harner , “Complications After Arthroscopic Knee Surgery,” American Journal of Sports Medicine 42 (2014): 292–296. 24284049 10.1177/0363546513510677

[4] R. M. Neagoe , S. Bancu , M. Muresan , and D. Sala , “Major Vascular Injuries Complicating Knee Arthroscopy,” Wideochir Inne Tech Maloinwazyjne 10 (2015): 266–274. 26240627 10.5114/wiitm.2015.52559 PMC4520854

[5] C. W. Ho , S. H. Lee , S. H. Wu , C. Y. Lin , C. H. Lee , and J. L. Wu , “Pseudoaneurysm Following Hamstring Tendon Harvest in Arthroscopic Anterior Cruciate Ligament Reconstruction: A Case Report,” BMC Musculoskeletal Disorders 21 (2020): 697. 33087086 10.1186/s12891-020-03721-4 PMC7579808

[6] J. D. Gliatis , S. L. Papagiannis , G. V. Sinos , E. D. Argyropoulou , and C. V. Kotsia , “Pseudoaneurysm of the Superior Lateral Genicular Artery of the Knee Following Arthroscopic Irrigation and Debridement and Review of Literature,” Indian Journal of Orthopaedics 57 (2023): 159–162. 36660486 10.1007/s43465-022-00790-6 PMC9789264

[7] J. Le Roux , M. Burger , G. Du Preez , and N. Ferreira , “The Reliability of Physical Examination in Diagnosing Arterial Injury in Penetrating Trauma to Extremities: A First Look at Different Anatomical Regions and Injury Mechanisms,” South African Medical Journal 111 (2021): 891–895. 34949255

[8] I. S. deSouza , R. Benabbas , S. McKee , et al., “Accuracy of Physical Examination, Ankle‐Brachial Index, and Ultrasonography in the Diagnosis of Arterial Injury in Patients With Penetrating Extremity Trauma: A Systematic Review and Meta‐Analysis,” Academic Emergency Medicine 24 (2017): 994–1017. 28493614 10.1111/acem.13227

[9] N. Runge , L. Hollifield , M. Arnold , and J. Oni , “Acute Popliteal Thrombus Following Total Knee Arthroplasty: A Case Report,” Medicine 99 (2020): e22500. 33080684 10.1097/MD.0000000000022500 PMC7571872

[10] M. S. Conte , A. W. Bradbury , P. Kolh , et al., “Global Vascular Guidelines on the Management of Chronic Limb‐Threatening Ischemia,” European Journal of Vascular and Endovascular Surgery 58 (2019): S1–S109.e33. 31182334 10.1016/j.ejvs.2019.05.006 PMC8369495

[11] K. Bernhoff and M. Bjorck , “Iatrogenic Popliteal Artery Injury in Non Arthroplasty Knee Surgery,” Bone & Joint Journal 97‐B (2015): 192–196. 10.1302/0301-620X.97B2.34353 25628281

[12] A. Nishimura , A. Fukuda , K. Kato , K. Fujisawa , A. Uchida , and A. Sudo , “Vascular Safety During Arthroscopic All‐Inside Meniscus Suture,” Knee Surgery, Sports Traumatology, Arthroscopy 23 (2015): 975–980. 10.1007/s00167-013-2774-7 24253374

[13] K. Bernhoff , K. Michaelsson , and M. Bjorck , “Incidence and Outcome of Popliteal Artery Injury Associated With Knee Dislocations, Ligamentous Injuries, and Close to Knee Fractures: A Nationwide Population Based Cohort Study,” European Journal of Vascular and Endovascular Surgery 61 (2021): 297–304. 33303313 10.1016/j.ejvs.2020.10.017

[14] T. I. Nicolino , J. Costantini , I. Astore , et al., “Incidence of Vascular Injury Associated With Knee Arthroplasty: Series of Cases,” European Journal of Orthopaedic Surgery & Traumatology 34 (2024): 3735–3742. 38252291 10.1007/s00590-023-03814-5

[15] A. Tarasiuk , R. S. Tubbs , N. Zielinska , P. Karauda , B. Gonera , and L. Olewnik , “Variations of the Popliteal Artery: A Review,” Annals of Anatomy 249 (2023): 152100. 37105405 10.1016/j.aanat.2023.152100

[16] D. Kim , D. E. Orron , and J. J. Skillman , “Surgical Significance of Popliteal Arterial Variants. A Unified Angiographic Classification,” Annals of Surgery 210 (1989): 776–781. 2589890 10.1097/00000658-198912000-00014 PMC1357871

[17] S. W. Kil and G. S. Jung , “Anatomical Variations of the Popliteal Artery and Its Tibial Branches: Analysis in 1242 Extremities,” Cardiovascular and Interventional Radiology 32 (2009): 233–240. 18982387 10.1007/s00270-008-9460-z

[18] B. Yanik , E. Bulbul , and G. Demirpolat , “Variations of the Popliteal Artery Branching With Multidetector CT Angiography,” Surgical and Radiologic Anatomy 37 (2015): 223–230. 25038837 10.1007/s00276-014-1346-y

[19] M. Bernard , M. Grothues‐Spork , A. Georgoulis , and P. Hertel , “Neural and Vascular Complications of Arthroscopic Meniscal Surgery,” Knee Surgery, Sports Traumatology, Arthroscopy 2 (1994): 14–18. 10.1007/BF01552648 7584170

[20] M. J. Matava , N. S. Sethi , and W. G. Totty , “Proximity of the Posterior Cruciate Ligament Insertion to the Popliteal Artery as a Function of the Knee Flexion Angle: Implications for Posterior Cruciate Ligament Reconstruction,” Arthroscopy 16 (2000): 796–804. 11078535 10.1053/jars.2000.18243

[21] A. Makino , M. Costa‐Paz , L. Aponte‐Tinao , M. A. Ayerza , and D. L. Muscolo , “Popliteal Artery Laceration During Arthroscopic Posterior Cruciate Ligament Reconstruction,” Arthroscopy 21 (2005): 1396. 16325093 10.1016/j.arthro.2005.08.028

[22] G. C. Lee , D. H. Kim , and S. H. Park , “Popliteal Artery Pseudoaneurysm After Anterior Cruciate Ligament Re‐Revision Using a Rigidfix Cross Pin,” Knee Surgery & Related Research 26 (2014): 121–124. 24944979 10.5792/ksrr.2014.26.2.121 PMC4061407

[23] M. E. Enríquez‐Vega , J. E. Cruz‐Castillo , E. Pacheco‐Pittaluga , H. Solorio‐Rosette , L. Linarte‐Marquez , and A. Iturburu‐Enríquez , “Vascular Injury as a Complication of Knee Arthroscopic Surgery. Report of Two Cases and Review of the Literature,” Cirugia y Cirujanos 81 (2013): 454–458. 25125066

[24] J. H. Yoo and C. B. Chang , “The Location of the Popliteal Artery in Extension and 90 Degree Knee Flexion Measured on MRI,” Knee 16 (2009): 143–148. 19046634 10.1016/j.knee.2008.10.009

[25] T. Mizuno , H. Hiraiwa , T. Tsukahara , et al., “Deep Flexion Helps to Avoid Popliteal Artery Injury During All‐Inside Lateral Meniscal Repair: A Cadaveric Study,” Knee 33 (2021): 159–168. 34624750 10.1016/j.knee.2021.09.004

[26] C. E. Franciozi , L. J. Albertoni , F. N. Ribeiro , et al., “A Simple Method to Minimize Vascular Lesion of the Popliteal Artery by Guidewire During Transtibial Posterior Cruciate Ligament Reconstruction: A Cadaveric Study,” Arthroscopy 30 (2014): 1124–1130. 25193126 10.1016/j.arthro.2014.07.003

[27] E. Alentorn‐Geli , J. J. Stuart , J. H. James Choi , A. P. Toth , C. T. Moorman, 3rd , and D. C. Taylor , “Posterolateral Portal Tibial Tunnel Drilling for Posterior Cruciate Ligament Reconstruction: Technique and Evaluation of Safety and Tunnel Position,” Knee Surgery, Sports Traumatology, Arthroscopy 25 (2017): 2474–2480. 10.1007/s00167-015-3958-0 26718637

[28] E. Alentorn‐Geli , J. J. Stuart , J. H. Choi , A. P. Toth , C. T. Moorman, 3rd , and D. C. Taylor , “Inside‐Out Antegrade Tibial Tunnel Drilling Through the Posterolateral Portal Using a Flexible Reamer in Posterior Cruciate Ligament Reconstruction,” Arthroscopy Techniques 4 (2015): e537–e544. 26900551 10.1016/j.eats.2015.05.016 PMC4722232

[29] V. Kp , J.‐R. Yoon , K. W. Nha , J.‐H. Yang , J.‐H. Lee , and H. Jegal , “Popliteal Artery Pseudoaneurysm After Arthroscopic Cystectomy of a Popliteal Cyst,” Arthroscopy 25 (2009): 1054–1057. 19732646 10.1016/j.arthro.2009.05.005

[30] D. W. Mao , U. Upadhyay , S. Thalanki , and D. Y. H. Lee , “All‐Inside Lateral Meniscal Repair via Anterolateral Portal Increases Risk of Vascular Injury: A Cadaveric Study,” Arthroscopy 36 (2020): 225–232. 31787348 10.1016/j.arthro.2019.07.023

[31] G. Fang , B. Chen , D. Q. Guo , et al., “Treatment of Popliteal Artery Aneurysm‐Induced Emergencies,” Chinese Medical Journal 133 (2020): 94–96. 31923111 10.1097/CM9.0000000000000575 PMC7028201

[32] J. S. Lee , S. C. Park , and S. D. Kim , “Management for Delayed and Symptomatic Pseudoaneurysm by Iatrogenic Popliteal Artery Injury During Posterior Cruciate Ligament Surgery,” Asian Journal of Surgery 43 (2020): 860. 32439298 10.1016/j.asjsur.2020.04.006

[33] Y. C. Chan , A. C. Ting , K. X. Qing , and S. W. Cheng , “Delayed Presentation of Popliteal Pseudo‐Aneurysm Following Soccer Football Injury,” Annals of Vascular Surgery 24 (2010): 553.e13–553.e16. 10.1016/j.avsg.2009.09.017 20097521

[34] K. Jagdish , M. Paiman , A. Nawfar , et al., “The Outcomes of Salvage Surgery for Vascular Injury in the Extremities: A Special Consideration for Delayed Revascularization,” Malaysian Orthopaedic Journal 8 (2014): 14–20. 10.5704/MOJ.1403.012 PMC4093557 25279079

[35] F. Fluck , A. M. Augustin , T. Bley , and R. Kickuth , “Current Treatment Options in Acute Limb Ischemia,” Röfo 192 (2020): 319–326. 31461761 10.1055/a-0998-4204

